# Antioxidant Effect of *Lycium barbarum* Leaf through Inflammatory and Endoplasmic Reticulum Stress Mechanism

**DOI:** 10.3390/antiox10010020

**Published:** 2020-12-28

**Authors:** So Rok Lee, Mi-Yeong An, Hye-Jeong Hwang, Ju-Gyeong Yoon, Jin Ah Cho

**Affiliations:** 1 Department of Food and Nutrition, Chungnam National University, 99, Daehak-ro, Yuseong-gu, Daejeon 34134, Korea; sj807sr@cnu.ac.kr (S.R.L.); miyeong1119@cnu.ac.kr (M.-Y.A.); jugyeongs2@gmail.com (J.-G.Y.); 2 Department of Agrofood Resources, National Institute of Agricultural Sciences, Rural Development Administration, Wanju 55365, Korea; hjh1027@korea.kr

**Keywords:** *Lycium barbarum*, Goji berry, antioxidant, anti-inflammatory, ER stress

## Abstract

Although the prevalence and incidence of inflammatory bowel disease (IBD), a defective immune response of the gastrointestinal tract, has been increasing in North America and Western Europe, recent studies have shown that this disease is also increasing rapidly in Asia. Several studies have been searching for functional foods that can prevent or reduce IBD symptoms because the drug treatments for IBD are expensive with complications. Genome-Wide Association Study (GWAS), an observational study of a genome-wide set of genetic variants in different individuals, showed that endoplasmic reticulum (ER) stress is one of the causes of IBD. Previously, we reported the effects of *Lycium* *barbarum* fruit and this study investigated the effects of *Lycium barbarum* leaf (LL) on inflammation and ER stress of the intestine. The paracellular permeability, antioxidant, and anti-inflammatory response were measured on polarized Caco-2 cells. The ER stress pathway and pro-inflammatory cytokines were evaluated on MEF-knockout cell lines, and on the intestines of the mice fed a high-fat diet with lipopolysaccharide-induced inflammation. Our data showed that the LL pretreatment strengthened the tight junction integrity and reduced NO production both in the presence and in the absence of inflammation. Furthermore, LL inhibited ER stress and inflammation via IRE1α and XBP1 in vitro as well as in the inflamed intestines of mice, highlighting the antioxidant and anti-inflammatory function of LL in an IRE1α-XBP1-dependent manner.

## 1. Introduction

The endoplasmic reticulum (ER) is in charge of the synthesis, folding, and processing of secretory and transmembrane proteins. ER stress is caused by the accumulation of misfolded or unfolded proteins in the ER, mostly caused by environmental factors such as reactive oxygen species (ROS), major inflammatory mediators in the disease pathogenesis [1]. Then, calcium-dependent protein folding enzymes release calcium from the ER lumen and increase the concentration of calcium influx into the cytoplasm, worsening the level of ER stress, oxidant reactions, and inflammatory reactions [2,3]. This process activates three endoplasmic reticulum transmembrane domains: IRE1, protein kinase RNA-like endoplasmic reticulum kinase (PERK), and ATF6. In the resting state, the molecular chaperone, BiP (78 kDa glucose control protein GRP78), a central regulator of ER stress, maintains IRE1, PERK, ATF6 in an inactive state by binding to their luminal domains. In the stressed state, BiP dissociates from these ER stress receptors and binds to an overwhelming misfolded protein, unfolded proteins. Then, BiP-free ER stress receptors become active. Activated PERK causes the translational attenuation of the protein machinery involving in the cell cycle and producing cell cycle arrest in the G1 phase [4]. Unsolved ER stress induces the unfolded protein response (UPR)-related genes, primarily chaperones, such as BiP, to prevent the further accumulation of unfolded/misfolded proteins [5,6]. Activated IRE1α activates an ER-related associated degradation (ERAD) system, consisting of ubiquitin-dependent proteasomes [7]. IRE1α is self-phosphorylated to activate the RNase activity, initiating the removal of the 26-base intron from the mRNA encoding X-box-binding protein 1 (XBP1) [8]. This results in translational frameshifting and translation of the XBP1 isoform with potent activity as a transcription factor, resulting in activation of the NF-kB pathway. Under the conditions of prolonged stress, the proapoptotic protein CHOP (CCAAT/enhancer-binding protein homologous protein) is upregulated causing a proapoptotic drive at the mitochondria [9]. 

Approximately 20–40% of inflammatory bowel disease (IBD) patients in Western countries are obese and it has been reported that there is correlation between the incidence of IBD and a high-fat diet rich in cholesterol and animal fat [10,11]. ER stress and UPR induction are critical to intestinal stem cells, and it has been reported that damage to UPR signaling leads to chronic inflammatory diseases such as IBD and irritable bowel syndrome (IBS) [12]. Goblet cells secrete mucin to form a protective luminal mucus layer and Paneth cells secrete defensin and other antimicrobial peptides to keep the crypt of the small intestine in a sterile state [13]. The depletion of goblet cells results in a failure to protect against and invasion of pathogenic bacteria is a common pathological phenomenon in IBD [10]. In addition, intestinal epithelial cells, which are functionally impaired in IBD, can trigger an inflammatory response [5].

*Lycium barbarum* (*L. barbarum*), also known as Goji berry, has attracted attention as a superfood in Western countries. It has been used mainly as an edible and traditional medicinal plant for a long time in Eastern countries, such as China and Korea [14]. *Lycium barbarum* contains many polysaccharides, flavonoids, and polyphenol compounds such as chlorogenic acid, p-coumaric acid, caffeic acid, ferulic acid and Gentisic acid [15]. While the fruits are widely used worldwide, their leaves are usually wasted. However, if any functional components or effects in the leaves were well studied with the clear mechanisms, it would be most appreciated in the field of agriculture. So far, the main active flavonoid of the *L. barbarum* leaf (LL) was identified as rutin [16]. Hypoglycemic, antimicrobial, antioxidant, anti-aging, anticancer and lipid-lowering effects have been reported [17,18,19,20]. However, little is known about its effects and the mechanism on the intestine. Previously, we investigated *L. barbarum* fruits extract and found that it enhanced the gut barrier function and reduced inflammation and ER stress. Therefore, this study investigated the antioxidant and anti-inflammatory effects of LL through the ER stress and oxidative stress pathway.

## 2. Materials and Methods 

### 2.1. Materials

The dried leaves of *L. barbarum* were purchased from Ningxi Hui in China. The leaves were extracted using 70% ethanol at room temperature for seven days. After filtration through filter paper Advantech No.3 (Toyo Roshi Kaisha, Japan), the extract was concentrated with a rotary evaporator A-1000S (EYELA, Tokyo, Japan) and used in the experiment. The leaf extract of *L. barbarum* was dissolved in 70% DMSO (Sigma-Aldrich Co., Saint louis, MO, USA) at 25 mg/mL to be used as the stock solution. 

### 2.2. Liquid Chromatography (LC)–Mass Spectrometry

LC-mass were performed SYNAPT G2 Si HDMS QTOF (Waters Corporation, Milrord, Massachusetts, USA). LC conditions were performed using a C18 column (ACQUITY UPLC^®^ HSS T3 C18, 1.8 µm, 2.1 × 100 mm, Waters, Worcester, MA, USA); the temperature at 35 °C with UV detector at 265 nm. The injection volume was set to 5 μL and analyzed. The mobile phase was analyzed using water (A) and methanol (B) diluted with 0.1% formic acid. As for the solvent gradient conditions, the initial flow of A and B at a ratio of 90:10 for 0 to 5 min, followed by 13 to 18 min of B 100%. After that, it was set to run for 18–20 min in the same way as the initial conditions. The ionization condition was set to positive, and the scan range was set to 50~1550 *m*/*z*. The source of the mass spectrometer was analyzed using electrospray ionization (ESI).

### 2.3. Cell Culture

Human intestinal epithelial cells, Caco-2, were purchased from the American Type Culture Collection (Manassas, VA, USA). The MEF cells were a generous gift from David Ron (University of Cambridge, Cambridge, UK). The cells were cultured in Dulbecco’s modified Eagle’s medium (Gibco, Grand Island, NY, USA) with 1% Penicillin-Streptomycin (Sigma-Aldrich Co., Saint louis, MO, USA) and 10% heat-inactivated fetal bovine serum (Gibco, USA) at 37 °C under an atmosphere containing 5% CO_2_. The Caco-2 cells were polarized on Transwell (SPL Life Sciences, Pocheon, Korea) coated with 396 μg/cm^2^ Type I collagen (Corning, VA, USA), in which monolayers were grown for 10 days. The electrical resistance was measured using trans epithelial electric resistance measurements (EVOM2, World Precision Instruments Inc., Sarasota, FL, USA). When the transepithelial/ transendothelial electrical resistance (TEER) reached 500–600 Ωcm^2^, the apical side of the cells was treated with LL and further stimulated with Thapsigargin (TG, Sigma-Aldrich Co., Saint louis, MO, USA) for 1 h or 4 h to induce ER stress or stimulated with cytokine cocktails (CT, 50 ng/mL TNFα + 50 ng/mL IFN-γ + 25 ng/mL IL1β + 10 μg/mL LPS) for 16 h to induce inflammation. 

### 2.4. Experimental Animals

#### 2.4.1. Mice

Five-week-old male BALB/c mice were purchased from DaHanBioLink Co., Ltd. (Eumseong, Korea). After one week of quarantine, the mice were fed a 60% high-fat diet (Research Diet, New Brunswick, NJ, USA) for 46 days. After 23 days, the mice were divided randomly into four groups (each group *n* = 8): HFD diet (H), HFD diet + LPS injection (HL), HFD diet + LPS injection + 150 mg/kg LL (HLL) oral gavage, and HFD diet + LPS injection + 300 mg/kg (HLH) oral gavage. The mice were administered 150 mg/kg and 300 mg/kg LL orally for 20 days daily. The mice were injected intraperitoneally (i.p.) with 5 mg/kg Lipopolysaccharides (LPS, InvivoGen, San Diego, CA, USA) and sacrificed after 1 hr. All animal experiments were approved by the Committee of Animal Care and Experiment of Chungnam National University (201909A-CNU-134) and were carried out in accordance with the National Institutes of Health Guide for the Care and Use of Laboratory Animals (NIH Publications No. 8023, revised 1978).

#### 2.4.2. Zebrafish

Zebrafish were maintained at 28 °C for 14 h in the light cycle and 10 h in the dark cycle. Brine shrimp were raised in the laboratory and fed to the fish four times a day. To collect the fertilized embryos, individual males and females were separated in a mating cage overnight and spawned in the next morning. To evaluate the toxicity to the zebrafish embryos, the fertilized eggs were washed five to six times with a solution of 4 ppm methylene blue in 0.1% egg water and washed again with a solution of 1 ppm in concentration. The washed fertilized eggs were observed under a microscope, and the normal fertilized eggs were then selected and used in the experiment. The selected fertilized eggs were placed 10 per well in a 24-well plate (Hyundai Micro Co., Seoul, Korea) and treated with 2 mL of different concentrations of LL (0, 12.5, 25, 50, 100, and 250 μg/mL) in 0.1% egg water. The egg water with different concentrations of LL was changed every 24 h. The developmental process and toxicity were evaluated at 2, 4, 8, 14, 22, 30, 48, and 75 h using a DM2000 (Leica Co., Wetzlar, Germany) and a SZ2-ILST microscope (Olympus, Tokyo, Japan). All experiments on the zebrafish were performed according to the protocol approved by the Animal Care and Use Committee of Chungnam National University (CNU-01027).

### 2.5. Cell Viability Assay

Water-soluble tetrazolium (WST) analysis was performed to determine the cell viability using an EZ-cytox (Daeil lap service Co. Ltd., Korea). The cells were pretreated with various concentrations of LL (0, 12.5, 25, 50, 100, and 250 μg/mL) for 24 h followed by the WST assay according to the manufacturer’s instructions. The absorbance was measured at 450 nm on the xMarkTM Microplate Absorbance Spectrophotometer (Bio-Rad Inc., Hercules, CA, USA) after 3 h.

### 2.6. Nitric Oxide (NO) Assay 

Polarized Caco-2 cells on transwell (SPL Life Sciences, Pocheon, Korea) were pretreated with LL (0, 12.5, 25, 50, and 100 μg/mL) for 24 h and stimulated with/without the cytokine cocktail (CT, 50 ng/mL TNFα + 50 ng/mL IFN-γ + 25 ng/mL IL1β + 10 μg/mL LPS) for a further 16 h to induce inflammation. IL1β and tumor necrosis factor-alpha (TNF-α) from JW Creagene Co. (Seongnam, Korea), IFN-γ from R&D Systems Inc. (Minneapolis, Minnesota, USA), and LPS from InvivoGen (San Diego, CA, USA) were purchased. NO production of the conditional media from the apical side of the chamber was measured using a Griess reagent system (Promega Co., Madison, WI, USA) according to the manufacturer’s instructions. The absorbance was measured at 540 nm using the xMark™ Microplate Absorbance Spectrophotometer (Bio-Rad Inc., Hercules, CA, USA). 

### 2.7. Paracellular Permeability

Polarized Caco-2 cells were pretreated with LL on the apical side for 24 h, and stimulated with/without CT for a further 16 h to induce inflammation. The apical and basal sides were washed with Hanks’ balanced salts (HBSS, Sigma-Aldrich Co., Poole, UK) supplemented with 10 mM Hydroxyethyl piperazine Ethane Sulfonic acid (HEPES, Sigma-Aldrich Co, Saint Louis, MO, USA). One mg/mL of 4 kDa Fluorescein isothiocyanate dextran (FITC—dextran, Sigma-Aldrich Co., Uppsala, SWEDEN) in a HBSS/HEPES solution was added to the apical side and incubated for 72 h. The absorbance of the HBSS/HEPES solution on the basal side was measured every 0, 1, 2, 3, 6, 24, 30, 48, and 72 h. The fluorescence signals were measured using a DTX 800 multimode detector (Beckman Coulter, Inc., Brea, CA, USA) at 490 nm for excitation and 520 nm for emission.

### 2.8. RNA Extraction and cDNA Synthesis

The total RNA was extracted from the cells. Briefly, after removing the cell medium, Tri-reagent (MRC Inc., Cincinnati, OH, USA) was added to each well, and the resulting lysates were harvested. Chloroform was then added, and the mixture was centrifuged at 12,000× *g* for 15 min at 4 °C. The supernatant was separated, mixed with isopropanol, and centrifuged at 12,000× *g* at 20 °C for eight minutes. The RNA pellets were harvested, and the RNA concentration was measured using Nano Drop ONE (Thermo Scientific Inc., Madison, WI, USA). The cDNA was synthesized using an RT-Kit (BioFACT Co., Daejeon, Korea).

### 2.9. Real-Time Polymerase Chain Reaction 

Real-time qPCR was performed with cDNA synthesized using 2X Real-time PCR Master Mix Kit (BioFACT Co., Daejeon, Korea). Experimental steps using real-time qPCR machine (Agilent Co., Palo Alto, CA, USA) are as follows: the denaturation step took 15 min at 95 °C; the denaturing step took 20 s at 95 °C; the Anneal and Extension step took 40 s at 60 °C; one amplification cycle was performed for 45 cycles. The data were analyzed using the Agilent AriaMX 1.0 program. Table 1 lists the primer sequences used in the study.

### 2.10. Reverse Transcription Polymerase Chain Reaction (RT-PCR)

RT-PCR was performed to measure the RNA expression of the target gene using the 2X Taq Basic PCR Master Mix2 (BioFACT Co., Daejeon, Korea). Table 2 lists the primer sequences used in the experiment. The PCR mixture was loaded on 3% agarose gel for 45 min and then exposed to UV Light using the AE-9000 E-graph (ATTO, Japan).

### 2.11. Enzyme-Linked Immunosorbent Assay (ELISA)

The blood of mice was collected by cardiac puncture after anesthesia and allowed to clot for 30 min at room temperature. The serum was then centrifuged at 1000× *g* for 10 min. The supernatant was collected to perform the ELISA assay for interleukin 6 (IL6) according to the manufacture’s instruction (BD Biosciences, Franklin Lakes, NJ, USA).

### 2.12. Tissue Histology

The colon tissues of mice were fixed in a 10% formaldehyde solution, processed into paraffin blocks using standard methods, and then they were sectioned. The tissue sections were stained with hematoxylin and eosin (H&E) and Periodic Acid-Schiff (PAS) (T&P bio, Gwangju, Gyeonggi-do, Korea), and all stained tissues were taken with an optical microscope (OLYMPUS, Tokyo, Japan).

### 2.13. Flow Cytometry for Cell Cycle

After the entire experiment was completed, the mice were sacrificed, and the spleens were isolated for the ex vivo splenocytes culture, as described previously [21]. For cell cycle analysis, the mouse splenocytes were minced and isolated using a cell strainer with 100 µm diameter pores (SPL Life Sciences, Korea) and washed twice with RPMI1640. The splenocytes were cultured in RPMI1640 media with 10% FBS, stimulated with 10 µg/mL of LPS for 72 h, and fixed with 70% ethanol. The cells were stained with a cell cycle kit (Millipore, Billerica, MA, USA) for 30 min at room temperature in the dark, vortexed gently, and read on the Muse Cell analyzer (Millipore, Darmstadt, Germany).

### 2.14. Statistical Analysis

Statistical processing of all experiments was analyzed using the SPSS/Windows 24.0 (SPSS Inc., Chicago, IL, USA) program. All experiments were conducted three or more times, and the experimental results are expressed as the mean ± standard deviation. A student’s *t*-test was used to examine the difference between the mean values between the two groups. One-way ANOVA was performed to analyze the difference between the mean values of three or more groups. After one-way ANOVA, the difference between the independent variables was confirmed using Duncan’s multiple range test, and the statistical significance was defined as being statistically significant when *p* < 0.05.

## 3. Results

### 3.1. LC-MS Analysis of L. barbarum Leaf 

**Figure 1:**
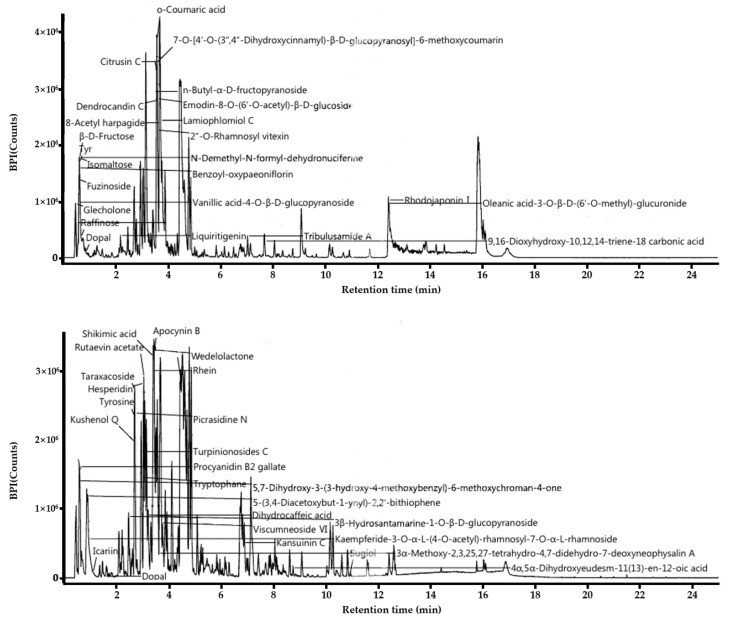
Functional components of *L. barbarum* leaf extract. Positive (**upper**) and negative (**down**) base peak intensity (BPI) chromatograms of the *L. barbarum* leaf was measured using ultra-performance liquid chromatography coupled with electrospray ionization/quadrupole-time-of-flight mass spectrometry.

In this study, the components of the ethanol extract of *L. barbarum* leaf were analyzed using LC/MS. Major compounds detected were mostly polyphenolic secondary metabolites, flavonoids (Figure 1). *O*-coumaric acid, Apocynin B, dendrocandin C, Citrusin C, 2-*O*-rhamnosyl vitexin, benzoyl oxypaeoniflorin, kushenol Q, shikimic acid, wedeloactone, rhein, dihydrocaffeic acid and Procyanidin B2 gallate were detected as major components related to anti-inflammatory and antioxidant. One of the phenolic acids, *O*-coumaric acid, a hydroxycinnamic acid found in vinegar, has been previously reported previously to inhibit cell growth in 3T3-L1 preadipocytes [22]. Apocynin, an inhibitor of NADPH oxidase, has been reported to prevent the production of the superoxide in human white blood cells or granulocytes, thus have beneficial effect in reducing ROS and the level of inflammatory cytokines in colitis models [23]. Dendrocandin C has been reported for potent antioxidant activity isolated from D. candidum stems [24]. Vitexin is an apigenin flavone glycoside with a variety of pharmacological effects, including antioxidant, anti-inflammatory, anticancer, antinociceptive, and neuroprotective effects [25]. Procyanidin B2 gallate, known to inhibit T-cell cytokines, is a polyphenol widely found in plants and has antioxidant and anti-inflammatory properties [26]. Since the antioxidant activity of polyphenols varies depending on the structure of the hydroxyl group, the gallate group may exhibit stronger antioxidant activity. Citrusin C is commonly found in common sage, lemon, herbs, and spices as phytochemical. Therefore, the major antioxidants were found in LL extract and this might be responsible for antioxidant and anti-inflammatory functions, which were examined further in this study below.

### 3.2. In Vitro and In Vivo Toxicity Assessment

**Figure 2:**
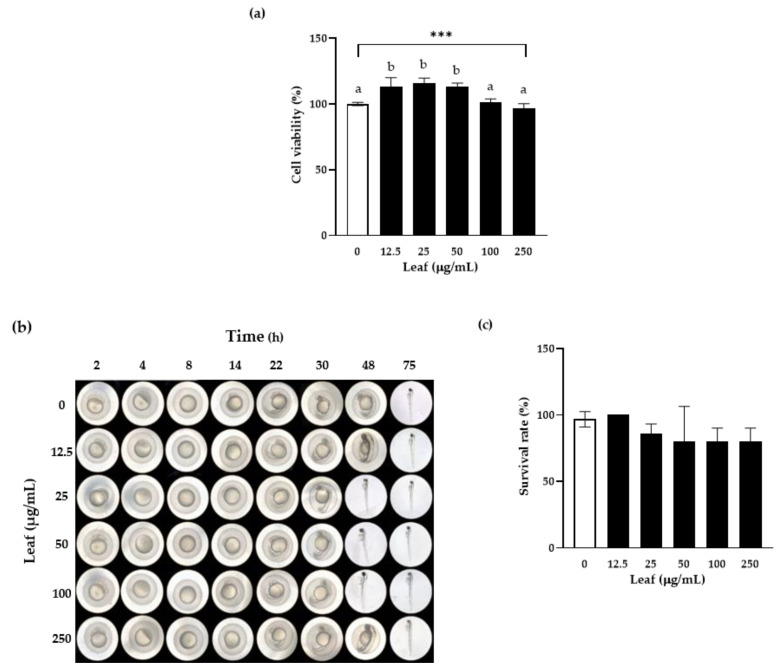
Effect of LL on cell viability in vitro and in vivo. Caco-2 cells were treated with the indicated concentrations of LL for 24 h, and the cell viability was measured using a WST assay (**a**). Zebrafish eggs were incubated with the different concentrations of LL in the egg water for up to 75 h, and photographs were taken (**b**). The survival rates of zebrafish eggs were calculated by counting the hatched eggs for 72 h (**c**). The white bar represents negative control set as 100%. Data are shown as the means ± SD of at least three independent experiments. Significant differences between different concentrations were analyzed using Duncan’s multiple range test with one-way ANOVA and values labeled with different letters are significantly different (*p* < 0.05). Significant different value compared to the control group was analyzed using a student’s *t*-test. *** *p* < 0.001. LL: *Lycium barbarum* leaf.

A WST assay was performed on human intestinal epithelial cells, Caco-2, to examine the effect of LL on cell viability. When the cells were treated with LL at different concentrations for 24 h, as shown in Figure 2a, the cell viability was significantly higher than 100% in the presence of LL up to 50 μg/mL, suggesting that LL might positively affect the proliferation of intestinal epithelial cells up to 50 μg/mL. Based on this result, the concentration used in all subsequent in vitro experiments was determined to be up to 250 μg/mL.

The Zebrafish (Danio rerio) shares almost 78% genetic sequences with humans. Hence, similar human gastrointestinal diseases can be induced and have been studied in zebrafish [27,28]. In addition, zebrafish eggs are used widely in research because of their transparency and ease of observing the development of internal organ structures. Therefore, in this study, the zebrafish were treated with various concentrations of LL and observed at the various time-points to examine the toxicity and the developmental effects of LL in vivo (Figure 2b). No structural disorders were observed during egg development with concentrations up to 250 μg/mL. On the other hand, hatching occurred faster at concentrations of 25–100 μg/mL compared to the negative control, which is the consistent result with the cell proliferation in vitro. Figure 2c showed no significant difference regardless of the LL concentrations, suggesting that LL does not affect the survival rate. In conclusion, LL does not exhibit toxicity up to 250 μg/mL in vitro and in vivo, and it appears that LL might have a positive effect on the proliferation of intestinal epithelial cells and the early development of zebrafish eggs.

### 3.3. Nitric Oxide (NO) Production and Paracellular Permeability of Polarized Caco-2 Cells

The tight junction (TJ) is the most important anatomical structure for maintaining the selective permeability and integrity of the intestinal epithelium. When local inflammation in the intestine occurs, the levels of inflammatory cytokines, such as IFNγ, IL1β, IL6, and TNFα rise, interfering with the barrier function by impairing the TJ integrity and allowing pathogens to penetrate the intestinal tissues easily.

**Figure 3:**
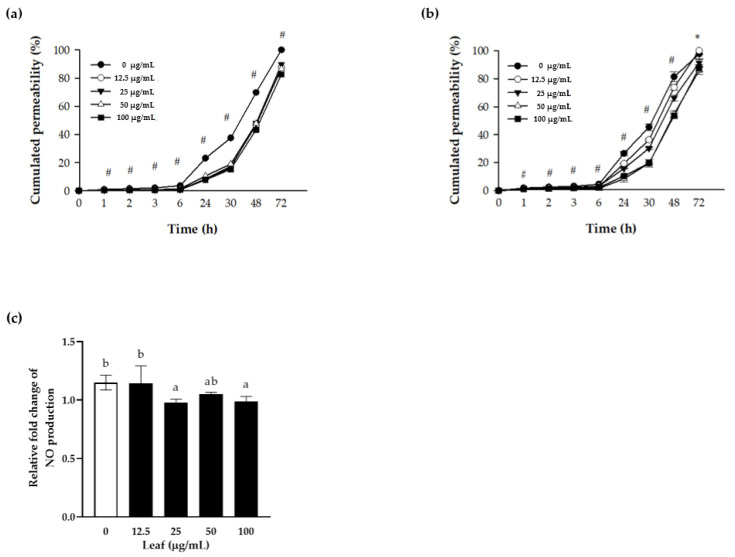
Protective effect of LL on the cell permeability. Polarized Caco-2 cells were pretreated with the indicated concentrations of LL for 24 h, followed by stimulation without (**a**) or with (**b**) cytokine cocktail (50 ng/mL TNF-α + 50 ng/mL IFN-γ + 25 ng/mL IL1β + 10 μg/mL LPS) apically for an additional 24 h. One mg/mL of FITC-dextran fluorescein was added to the apical side, and the absorbance was measured at the indicated time point in the conditional media from the basal side. Before adding FITC-dextran, the conditional media from the apical side were harvested for the NO assay to measure NO production (**c**). The white bar represents negative control set as 1.0. Data are shown as the means ± SD of at least three independent experiments. Significant differences between different concentrations were analyzed using Duncan’s multiple range test with one-way ANOVA and values labeled with different letters are significantly different (*p* < 0.05). Significant different value compared to the control group was analyzed using a student’s *t*-test.* *p* < 0.05, # *p* < 0.001. LL: *Lycium barbarum* leaf.

Polarized Caco-2 cells have functions and structures similar to those of intestinal epithelium, which are useful for observing the changes in permeability. In the current experiment, the apical side of polarized Caco-2 cells was pretreated with LL for 24 h in the presence or absence of cytokine cocktail for an additional 24 h. Subsequently, FITC-dextran was added to the apical side, and the absorbance was measured from the conditional media of the basal side to investigate the permeability of TJ over time. FITC-dextran on the apical side permeated significantly less in the LL-treated cells compared to the control cells. The level of accumulated permeation on the basal side of the cell layer increased with increasing LL concentration (Figure 3a). In the presence of inflammation represented as cytokine cocktail stimulation, the LL-treated cells showed a significant and concentration-dependent reduction of permeated FITC-dextran, suggesting the protective effect of LL on intestinal epithelium barrier both in the normal state or inflamed state of the intestine (Figure 3b). In summary, LL maintains the TJ integrity tightly, regardless of the presence or absence of external inflammatory stimuli on polarized Caco-2 cells, suggesting that LL improves the function of the barrier.

Therefore, it was examined whether LL regulates NO production of polarized Caco-2 in the presence of the inflammation. The conditioned media were collected and the level of NO were calculated by fold change in the presence over the absence of cytokine cocktail stimulation (Figure 3c). Compared to the negative control, higher than 25 μg/mL LL reduced NO secretion to the conditioned media significantly, suggesting that LL inhibits NO production induced by inflammation.

### 3.4. Inhibitory Effect of LL on Inflammatory Response and XBP1 Splicing In Vitro

**Figure 4:**
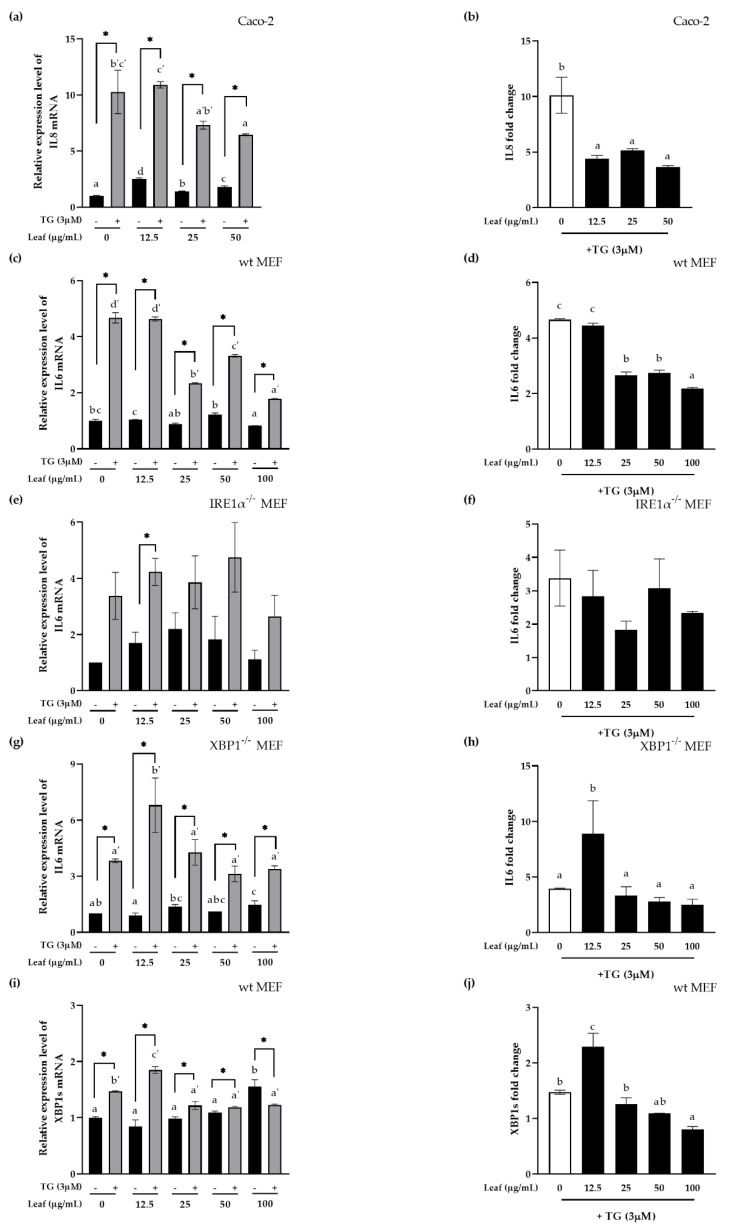
Anti-inflammatory effects of LL on the pro-inflammatory response induced by ER stress in XBP1 dependent manner. Polarized Caco-2 cells (**a**,**b**), wt MEF cells (**c**,**d**), IRE1α KO MEF (**e**,**f**), XBP1 KO MEF (**g**,**h**) were pretreated apically with the indicated LL concentrations for 24 h followed by 3 μM TG stimulation apically for an additional 4 h (**a**–**h**). Wt MEF cells (**i**,**j**) were pretreated apically with the indicated LL concentrations for 24 h followed by 3 μM TG stimulation apically for an additional 1 h. The cDNA from the cells were prepared for real-time qPCR targeting the genes as indicated and normalized by human GAPDH for Caco-2 or mouse β-actin for MEF. The fold change in target genes expression by TG was calculated at each LL concentration (**b**,**d**,**f**,**h**,**j**). The white bar represents negative control. Data are shown as the means ± SD of at least three independent experiments. Significant differences between different concentrations were analyzed using Duncan’s multiple range test with one-way ANOVA and values labeled with different letters are significantly different (*p* < 0.05). Significant different value compared to the control group was analyzed using a student’s *t*-test. * *p* < 0.05. LL: *Lycium barbarum* leaf, TG: Thapsigargin.

A previous study reported that XBP1 links ER stress and inflammation in IBD patients [29]. Therefore, this study examined whether XBP1 is a mechanistic linker between ER stress and the pro-inflammatory response in terms of the function of LL. Human IL8 or mouse IL6 (pro-inflammatory cytokines) mRNA expression or XBP1 splicing (XBP1s, a representative factor of ER stress) were normalized using human GAPDH or mouse β-actin in polarized human intestinal epithelial Caco-2 cells (Figure 4a,b), or the knockout (KO) mouse embryonic fibroblast (MEF, Figure 4c–j) cell lines, and all experiments were carried out at least three times. Cells were pretreated with LL for 24 h. ER stress was induced with 3 μM Thapsigargin (TG), a selective sarcoplasmic/ER Ca^2+^-ATPase (SERCA) inhibitor, which depletes intracellular Ca^2+^ storage and increases the extracellular Ca^2+^ influx through cell membrane-related channels, thereby raising the level of Ca^2+^ in the cytoplasm. IL8 or IL6 mRNA expression was increased significantly by the TG treatment compared to the negative control, indicating that inflammation was induced by ER stress in both Caco-2 cells (Figure 4a) and MEF cells (Figure 4c). When the cells were treated with LL, however, ER stress-induced IL8 or IL6 mRNA expression was decreased significantly compared to the negative control in both cell lines. Therefore, the fold change of IL8 or IL6 mRNA expression induced by ER stress were significantly decreased with the pretreatment of LL starting from the concentration of 25 μg/mL, indicating the anti-inflammatory effect of LL (Figure 4b,d). 

In the absence of IRE1α using IRE1α^−/−^ MEF cell lines, IL6 were induced by TG even in the absence of IRE1α, but not significantly with or without LL pretreatment except 12.5 μg/mL of LL (Figure 4e). The fold change of IL6 induction by LL was not changed significantly in the absence of IRE1α (Figure 4f) while it was significantly reduced by LL in the presence of IRE1α shown in Figure 4d, suggesting that IRE1α is the mediator of pro-inflammatory response inhibition in the mechanism of LL action.

Next, we examined the effect of LL in the absence of XBP1 using XBP1^−/−^ MEF cell. On the contrary, in the absence of XBP1, TG induced IL6 mRNA expression at all LL concentrations, suggesting that XBP1 is not the only bottleneck molecule to induce inflammation via ER stress as expected (Figure 4g). However, the fold change in IL6 expression by the TG treatment was not changed in the presence of different concentrations of LL compared to its absence except for 12.5 μg/mL of LL (Figure 4h). Therefore, TG-induced IL6 induction depends on the presence of XBP1. Note that wt MEF showed the inhibitory effect of LL dose-dependently on IL6 mRNA expression (Figure 4d). In conclusion, LL inhibits ER stress-induced proinflammatory response via IRE1α-XBP1 pathway.

Since XBP1s is the result of the cleavage of XBP1 mRNA by IRE1α endonuclease activity when IRE1α is activated by ER stress, next we confirmed XBP1 splicing in wt MEF whether XBP1 splicing by ER stress is inhibited by LL. TG significantly increased the expression of XBP1s mRNA in wt MEF cells, suggesting that TG induced XBP1 splicing. In addition, the expression of XBP1s mRNA by LL itself, even in the absence of ER stress, appeared to increase dose-dependently, even though it was not statistically significant except at the highest concentration. However, at 100 μg/mL TG-induced XBP1s expression were even significantly smaller than in the absence of TG, suggesting significant inhibition of XBP1 splicing effect of LL at the high concentration. When the fold change of XBP1s expression by the TG treatment were calculated by different concentrations of LL, ER stress-induced XBP1s were inhibited significantly by LL except for 12.5 μg/mL (Figure 4j). 

In summary, LL has an inhibitory effect of inflammation and XBP1 splicing in the presence of ER stress on polarized Caco-2 cells and wild type MEF cells. These data suggest that IRE1α-XBP1 is the major critical linker for inflammation-induced by ER stress in the mechanism of the anti-inflammatory function of LL.

### 3.5. Inhibitory Effect of LL on Inflammatory Response and ER Stress In Vivo 

To confirm the inhibitory effects of LL on inflammation and ER stress from in vitro result, HFD was used as a negative control for chronic mild inflammation, and LPS was used as an acute boost of the inflammation. All the mice were fed a high-fat diet (HFD) for 23 days and sequentially, two groups were orally gavaged with 150 mg/mL LL (HLL) or 300 mg/mL LL (HLH) daily for next 20 days with the continuation of high-fat diet feeding. At the end of the experiment, 5 mg/mL LPS were injected i.p. to the mice 1 h before the scarifying to boost inflammation except for the control group (H). The serum, small intestine, and the colon were collected and used for further analysis. The small intestine and colon tissue were homogenized, and the total RNA and cDNA were synthesized for RT-PCR and real-time qPCR, respectively. 

**Figure 5:**
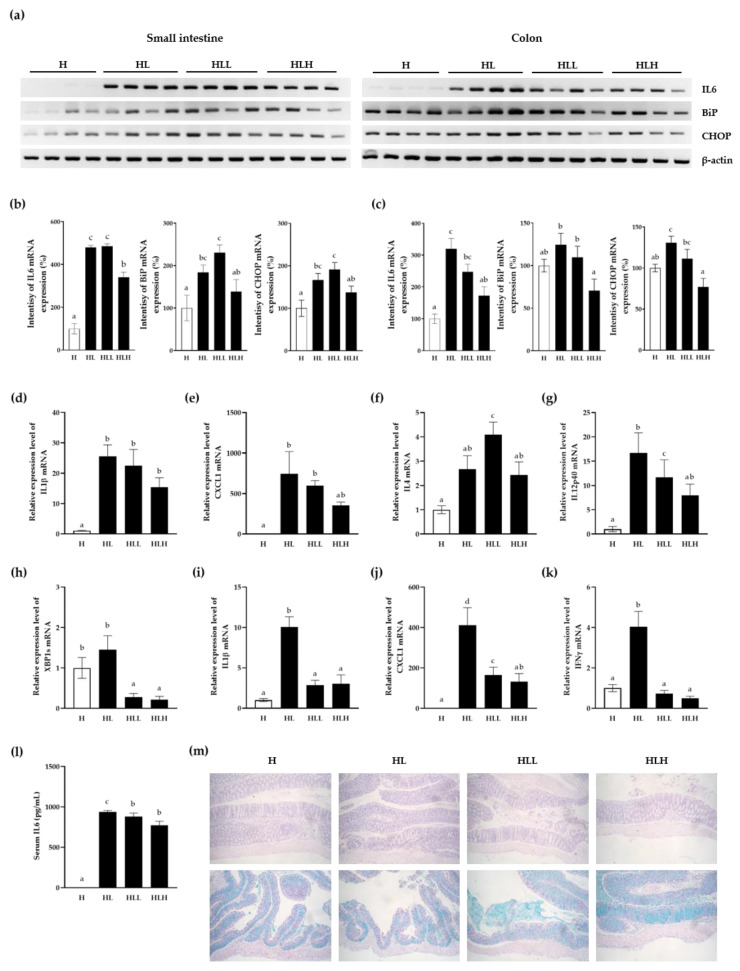
Anti-inflammatory effect of LL in the small intestine of the mice. The mice were fed with HFD alone for 23 days. Subsequently, 150 mg/kg and 300 mg/kg LL were administered daily for the next 20 days, along with HFD. Mice were i.p. with a 5 mg/kg of LPS followed by sacrifice after 1 h. The small intestine (**a**,**b**,**d**–**g**), the colon (**a**,**c**,**h**–**k**) and serum (**l**) were harvested for RT-PCR (**a**–**c**), real-time qPCR (**d**–**k**) and ELISA (**l**). β-actin was used as the loading control. The data are shown as the means ± SD of three independent experiments. The colons were harvested and stained with H&E and PAS (**m**). The white bar represents negative control. Data are shown as the means ± SD of at least three independent experiments. Significant differences between different groups were analyzed using Duncan’s multiple range test with one-way ANOVA and values labeled with different letters are significantly different (*p* < 0.05). HFD diet; H, HFD diet + LPS; HL, HFD + LPS + 150 mg/kg LL; HLL, HFD + LPS + 300 mg/kg; HLH.

First, the pro-inflammatory marker (IL6), ER stress hallmark (BiP), and ER stress-induced apoptosis marker (CHOP) were examined in the whole tissue of the small intestine and the colon by RT-PCR (Figure 5a) and quantified intensity graph (Figure 5b,c). The high concentrations of LL treated group (HLH) showed significantly lower IL6, BiP, and CHOP levels on the small intestine than the control group with HFD and LPS injection (HL). However, low dose of LL (HLL) did not affect the IL6 or CHOP mRNA levels, even though the BiP level on the small intestine was significantly higher than the HL group, which requires further mechanistic study for low-dose of LL. The colon of both low and high concentration of the LL treated groups showed a significant decrease in the inflammation and ER stress (IL6, BiP, and CHOP) compared to the HL group, suggesting the inhibitory effect of LL on inflammation and ER stress in the colon of the mice even when systemic inflammation was induced by LPS injection (Figure 5a,c). 

Next, the pro-inflammatory cytokines were examined in the whole tissue of the small intestines. The pro-inflammatory cytokine, IL1β, was increased significantly in HL group as shown in Figure 5d, and this phenotype was reduced significantly by a high dose of LL (HLH) (*p* = 0.001). The same phenomena occurred with the pro-inflammatory cytokine and chemokine (C-X-C motif) ligand 1 (CXCL1) (*p* = 0.006) (Figure 5e). IL4 has many biological roles, including the stimulation of activated B-cell and T-cell proliferation, the differentiation of B cells into plasma cells and the differentiation of naive helper T cells to Th2 cells as a key regulator in humoral and adaptive immunity. IL4 induces B-cell class switching to IgE and upregulates MHC class II production, which is associated with allergies. At the same time, IL4 decreases the production of Th1 cells, macrophages, interferon-gamma (IFNγ), and dendritic cells. Therefore, the significant decrease in IL4 in the HLH group compared to the HL group (*p* = 0.008) suggests that LL might reduce the transition of the chronic inflammation process from the innate to adaptive immunity, which also might involve the allergies (Figure 5f). In contrast to IL4, IL12, a bundle of IL12a (p35) and IL12b (p40), is involved in the differentiation of naive T cells into Th1 cells and stimulated the production of IFNγ and TNFα from T cells and natural killer (NK) cells, and reduced the IL4-mediated suppression of IFNγ. In the present study, LL produced a significant decrease in IL12p40 (*p* = 0.015), suggesting the inhibition of inflammation in the small intestine (Figure 5g).

Interestingly, when XBP1 was sliced by HFD and LPS treatment in the colon, LL inhibited XBP1 slicing significantly in a dose-dependent manner compared to even both the HL group and the HFD diet only, suggesting that LL positively affects the inflamed colon compared to the small intestine by inhibiting ER stress (Figure 5h). The pro-inflammatory cytokines were next examined in the whole tissue of the colon. The major pro-inflammatory cytokine, IL1β, CXCL1, and IFNγ, were increased significantly with HFD + LPS stimulation (Figure 5i–k). This phenotype was reduced significantly by LL in a dose- dependent manner. IFN-γ is the intermediate linker between the innate and adaptive immunity. The data suggest that LL might inhibit the development of immunity toward the adaptive immune response when exposed to a longer period for inflammation in the colon.

The serum IL6 level was also examined to identify systemic inflammation, showing increased serum IL6 by LPS, and its effects were inhibited significantly by the LL treatment (Figure 5l). These results suggest that LL inhibits the pro-inflammatory cytokines in the circulatory system.

The epithelium tissues of the colons were stained with H&E stain and PAS stain (Figure 5m). H&E stain showed no tissue damage of any difference on the anatomy of the colon due to HFD nor LPS stimulation as well as LL treated groups. Blue color by PAS stain showed the mucin protein in the colon. HFD and LPS stimulated group showed less mucin secretion compared to HFD only group and this phenotype were abolished by LL treatment showed significantly increased amount of mucin and even mucin layer on the epithelium. This data shows that LL might protect the intestine by enhancing the secretion of mucin and it would be interesting to follow up the mechanism of LL for stimulating mucin secretion in the future.

### 3.6. LPS-Induced Splenocytes Proliferation In Vivo

**Figure 6:**
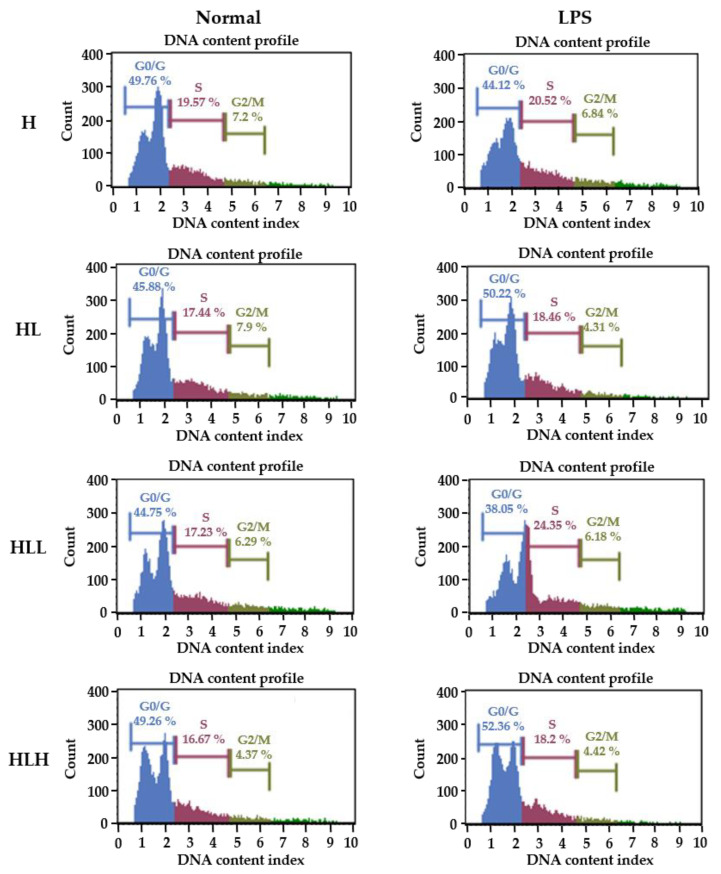
Effect of B lymphocyte activation of splenocytes. The mice were fed a HFD alone for 23 days. They were then administered 150 mg/kg and 300 mg/kg LL for the next 20 days daily along with an HFD. The mice were administered 5 mg/kg of LPS i.p. followed by sacrifice after 1hr. The spleens were isolated, and the splenocytes were isolated using a cell strainer and stimulated with 10µg/mL of LPS for 72 h, and fixed with 70% ethanol. The cells were stained for the cell cycle and analyzed using the Muse Cell analyzer. H, HFD diet; HL, HFD diet + LPS; HLL, HFD + LPS + 150 mg/kg LL; HLH, HFD + LPS + 300 mg/kg.

To confirm the inhibitory activity of LL on the adaptive immunity, the splenocytes from the mice used in the above experiments were isolated to determine if the LL intake affected the LPS-induced B lymphocyte proliferation/cell cycle (Figure 6). As shown in Table 3, in the unstimulated status, there were no differences in the splenocytes between the groups in G0/G1 arrest. In contrast, the HLL group showed a significant decrease in the S phase, and HLH showed a significant decrease in the DNA replication S phase and proliferation phase G2/M phase, suggesting that the oral intake of LL decreases the proliferation of splenocytes itself in vivo. On the other hand, with LPS stimulation to differentiate splenocytes to B lymphocyte because LPS is a B lymphocyte-activating factor, the HL group showed a significant increase in G0/G1 cycle arrest, decrease in the S phase and G2/M phage compared to the H group, as expected. In contrast, the HLL group reduced G0/G1 arrest and increased the S phage and G2/M phage, suggesting that the intake of low dose LL enhances B cell proliferation for adaptive immune response. High LL doses, however, affected the splenocytes in the opposite direction. These results suggest that the low dose intake of LL enhances B lymphocyte activation, while the high dose intake of LL suppresses B lymphocyte activation, suggesting that the low dose intake of LL might activate the long-term immunity of the host, such as allergies in this study. Note that the small intestine of the low dose LL treatment group showed a high level of IL4, which might mediate the allergies. Therefore, it would be interesting to determine if different LL concentrations should be considered to have the maximum positively enforced effect in this study.

## 4. Conclusions

*Lycium barbarum,* also known as Goji berry, contains many phenolic compounds, such as rutin, chlorogenic acid, *O*-Coumaric acid, caffeic acid, ferulic acid and gentisic acid. This study showed the antioxidant function of *Lycium barbarum* leaf (LL) related anti-inflammation with the ER stress mechanism in vitro and in vivo. Although rutin is known to be a main antioxidant component of *Lycium barbarum*, many other compounds related to anti-oxidant functions were found in *Lycium barbarum* leaf. It contained many polyphenolic compounds such as *O*-coumaric acid, apocynin B, dendrocandin C, citrusin C, 2-O-rhamnosyl vitexin, benzoyl oxypaeoniflorin, kushenol Q, shikimic acid, wedeloactone, rhein, dihydrocaffeic acid and procyanidin B2 gallate. They seem to be individually, synergically or additively worked more likely by different mechanisms to achieve the antioxidant and anti-inflammatory function examined in our study. Interestingly, LL inhibited inflammation mediated by an IRE1α-XBP1-dependent ER stress pathway, which might be linked to its antioxidant activity. On the other hand, only a high dose of LL might suppress the adaptive immune response represented as allergies in this study, which might involve PERK pathway related to cell apoptosis and arrest of the cell cycle. In conclusion, a proper concentration of LL intake would be beneficial to prevent excessive adaptive immunity and inflammation related to the ER stress, and it would be worth developing as a functional food to enhance gut health.

## Figures and Tables

**Table 1:** Real-Time qPCR primer sequences.

Gene	Accession Number	Primer	Sequence (5′ → 3′)
**Human**			
GAPDH	NM_001357943.2	Forward	ATG GGG AAG GTG AAG GTC G
		Reverse	GGG GTC ATT GAT GGC AAC AAT A
IL8	NM_001354840.3	Forward	GGG GTC ATT GAT GGC AAC AAT A
		Reverse	CAT GAA GTG TTG AAG TAG ATT TGC TTG
**Mouse**			
β-actin	NM_007393.5	Forward	TAC CAC CAT GTA CCC AGG CA
		Reverse	CTC AGG AGG AGC AAT GAT CTT GAT
XBP1s	NM_001271730.1	Forward	ACA CGC TTG GGA ATG GAC AC
		Reverse	CCA TGG GAA GAT GTT CTG GG
IL6	NM_001314054.1	Forward	CTG CAA GAG ACT TCC ATC CAG
		Reverse	AGT GGT ATA GAC AGG TCT GTT GG
IL1β	NM_008361.4	Forward	GAAATGCCACCTTTTGACAGTG
		Reverse	TGGATGCTCTCATCAGGACAG
CXCL1	NM_008176.3	Forward	ACT GCA CCC AAA CCG AAG TC
		Reverse	TGG GGA CAC CTT TTA GCA TCT T
IL4	NM_021283.2	Forward	ACA GGA GAA GGG ACG CCA T
		Reverse	GAA GCC CTA CAG ACG AGC TCA
IL12p40	NM_001303244.1	Forward	AGC AGT AGC AGT TCC CCT GA
		Reverse	AGT CCC TTT GGT CCA GTG TG
IFNγ	NM_008337.4	Forward	TCA AGT GGC ATA GAT GTG GAA GAA
		Reverse	TGG CTC TGC AGGATTTTCATG

**Table 2:** Reverse transcription PCR primer sequences (mouse).

Gene	Accession Number	Primer	Sequence (5′ → 3′)	Base Pair (bp)
β-actin	NM_007393.5	Forward	TCTCCAGCAACGAGGAGAAT	348
		Reverse	TGTGATCTGAAACCTGCTGC	
IL6	NM_001314054.1	Forward	CCGGAGAGGAGACTTCACAG	421
		Reverse	GGAAATTGGGGTAGGAAGGA	
BiP	NM_001163434.1	Forward	CTG GGT ACA TTT GAT CTG ACT GG	398
		Reverse	GCA TCC TGG TGG CTT TCC AGC CAT TC	
CHOP	NM_007837.4	Forward	CAC ATC CCA AAG CCC TCG CTC TC	286
		Reverse	TCA TGC TTG GTG CAG GCT GAC CAT	

**Table 3:** Percentage cell counts in a different phase of the cell cycle.

Group.	Percentage of Cell Counts in Different Phases (%)					
	Non-Stimulation			LPS Stimulation		
	G0/G1	S	G2/M	G0/G1	S	G2/M
H	48.47 ± 3.28	19.08 ± 0.95 ^b^	5.73 ± 0.66 ^b^	46.14 ± 1.89 ^a^	20.02 ± 1.46 ^a^	6.5 ± 0.7 ^c^
HL	48.47 ± 2	18.47 ± 1.5 ^b^	5.25 ± 0.62 ^b^	47.27 ± 2.43 ^ab^	18.63 ± 1.65 ^ab^	5.25 ± 0.68 ^ab^
HLL	48.47 ± 3.7	16.23 ± 1.9 ^a^	5.01 ± 1.09 ^b^	44.99 ± 4.59 ^a^	19.92 ± 2.44 ^b^	5.8 ± 0.78 ^bc^
HLH	48.47 ± 2.3	16.13 ± 1.5 ^a^	4.26 ± 0.54 ^a^	49.99 ± 3.19 ^b^	16.73 ± 2.1 ^b^	4.64 ± 1.12 ^a^

Data are shown as the means ± SD of at least three independent experiments. Significant differences between different groups were analyzed using Duncan’s multiple range test with one-way ANOVA and values labeled with different letters are significantly different (*p* < 0.05). HFD diet; H, HFD diet + LPS; HL, HFD + LPS + 150 mg/kg LL; HLL, HFD + LPS + 300 mg/kg; HLH.
